# Therapeutics, Ets.

**Published:** 1854-04

**Authors:** 


(Translated from the "Revue de Therapeutic Medico Chirurgicale.")

*Therapeudics. The Wonderful Effects of Belladonna in the Third Stage of Pneunomia.*

Case—On the 2d Oct. 1852, towards noon, M. X., a monk at Berguac, 25 years of age, pale, dark complexion, of a lymphatic sanguine temperament—of strong constitution, enjoying general good health, was imprudent enough, after inordinate exertions in the duties of his profession, to take, at a single draught, a large glass of iced water. During the night he was taken with a chill—called at six o'clock on the evening of the 3d to see him—found the following symptoms: anxiety, severe headache, eyes injected, white tongue, mouth open, intense thirst, nauseau, sleeplessness, urine scant with sediment, burning dry skin, full, frequent pulse, (115) dyspepnœa, painful persistent cough, *tearing* expectoration of viscid mucous, mixed with air and red blood—severe pain in the right lung, chiefly under the nipple—dullness over the inferior two-thirds of the lung of that side, with loud pneumonic crepitation. In spite of very low diet, appropriate drinks, rest, four free bleedings from the arm, (all more or less cupped,) within the space of thirty-six hours from my first visit, in spite of several large doses of tartar-emetic and ipecacuanha, two leechings, one to the painful part, the other around the inside of the ancles; in spite of blisters on the breast, and on the legs, synopism, emollient elysters, etc., the disease continued to progress.

At 10 o'clock, on the evening of the 9th, the local and general symptoms had continued to increase in number and gravity, until it was obvious that red hepatization of the lungs had taken place; face pale and anxious; extreme debility; lying all the time on the back; delirium—subsultus tendinum, vague expression, stupor, dry tongue; insatiable thirst for cold water; dry skin; eyes partly open; the eyeballs turned up; the pupils dilated; the cetina slightly sensible to strong light; passing urine involuntarily; pulse low, interrupted and diaphragmatic; respiration hard; cough scant and difficult expectoration, sputa thick and discolored; dullness increased and extended; respiratory murmnr, well over the seat of the disease; *rales* and other bronchial sounds; cold extremities; tendency to sinking towards the food of the bed.

Under the above circumstances, with no reasonable hope of evading an unhappy termination by ordinary means, we prescribed 15 centigrammes of the augueous extract of Belladonna, mixed ten grammes of "sirop de cappillaire," taken at one time.

At the end of an hour and a half the effect of the Belladonna was obvious, the patient slept tranquilly and quietly. In a few minutes his temperature augmented, face became adimated, warm, vapory, and anundant perspiration covered the body; the pulse rose, became stronger and less frequent; respiration was relieved; the symptoms all became more favorable. After two consecutive hours of repose, the patient awoke free from delirium, and feeling much better.

At 10 o'clock in the morning the pneumonia, which had to a certain degree re-assumed the characteristics of the first stage, was in a fair way to terminate by resolution.

Convalescence continued up to the 16 of November, when it was complete.—*De La Rue.*

